# Evaluation of breeding practices and morphological characterization of donkeys in Blouberg Local Municipality, Limpopo province: Implication for the design of community-based breeding programme

**DOI:** 10.1371/journal.pone.0278400

**Published:** 2022-12-14

**Authors:** Masixole Maswana, Thinawanga Joseph Mugwabana, Thobela Louis Tyasi

**Affiliations:** Department of Agricultural Economics and Animal Production, School of Agricultural and Environmental Sciences, University of Limpopo, Limpopo, South Africa; Universidade Federal de Mato Grosso do Sul, BRAZIL

## Abstract

Identification of livestock farmers’ breeding practices and morphological characterization of livestock are the important first steps to the successful implementation of any breeding programme. Community-based breeding programme (CBBP) has gained attention as a promising method for the genetic improvement of livestock but lacks information on donkey breeding. The study was conducted to identify donkey farmers’ breeding practices and donkey morphological characterization in three purposely selected villages (Thorne, Archibalt and Genau) located in Blouberg Local Municipality, Limpopo province of South Africa. Questionnaire survey method was used to collect the data from twenty-one donkey farmers while body weight and seventeen morphological traits were measured for the morphological characterization from seventy-four donkeys. Descriptive statistics and index values were computed to describe donkey breeding practices. Chi-square statistics were used to compare categorical variables among villages. General Linear Model procedure was used to compare morphological characteristics of donkeys among villages. The results revealed that there was no statistical different (P>0.05) observed on socio-economic characteristics of donkey farmers except on education level (P<0.05) among villages. Purpose of keeping donkeys, type of donkey breed kept, donkey coat colours, mating systems, reasons for culling, mating seasons and selection criterions were not significantly different (P>0.05) among villages. The most common trait preferences of donkey farmers among the surveyed villages were body size and growth rate in breeding males while in breeding females were body size, twining ability and mothering ability. The results indicated that in all the eighteen measured traits only four showed a significant difference (P<0.05) among the villages and some were significantly correlated with body weight (P<0.05). The results of this study will serves as basis for the development and implementation of CBBPs for donkey farmers at Blouberg Local Municipality of South Africa.

## Introduction

Donkeys have excellent draft power abilities, disease resistance, stress tolerance and can survive better under drought conditions than any other livestock species, due to their body structure and low dry matter intake requirements, which minimizes their water and maintenance needs in arid and semi-arid areas, due to their ability to survive on poor quality minimally supplemented feeds [1,2]. Donkeys continue to play a significant role in power-required activities in both rural and urban areas of developing countries, where donkeys are commonly employed for transportation of goods and movement of individuals from one location to another at extremely low prices [3,4]. Despite all the importance of donkeys in the community, they have been seen as less valuable by society because the government has not actively marketed them in comparison to other livestock [5]. Thus, their productivity is generally low as compared with other livestock in the community. However, community-based breeding programme (CBBP) might be the best method to improve the productivity of donkeys. CBBP is the method of breeding that involves a bottom-up approach where livestock specialists aid farmers to recognize their production challenges before the implementation of improvement program [6,7]. Knowing of farmers breeding objectives, trait preferences and selection criterions and also the characterisation of animal morphological traits helps in the designing of CBBP [8]. Morphological characterisation of animals is the first step to sustaining the use of a genetic resource [9]. Identification of farmers’ breeding practices, trait preferences and selection criterions have been investigated in goats [10], sheep [11], cattle [12,13] and chickens [14] for basis of designing and implementing the CBBP but limited in donkey farmers. Morphological characterization of donkeys has been conducted in different countries such as Algeria [15], Nigeria [16], Italy [17], Zimbabwe [18] and Turkey [19] but limited in South Africa. Hence, the objective of the current study was to identify donkey farmers’ breeding practices and also donkey morphological characterization of donkeys in three selected villages of Blouberg Local Municipality, Limpopo province of South Africa.

## Materials and methods

### Ethical approval

Ethical approval was granted by the University of Limpopo Animal Research Ethics Committee (ULAREC) with the number AREC/06/2021:PG before the commencement of the study.

### Study site

The study was carried out in the Blouberg Local Municipality, which is part of the Capricorn District Municipality in Limpopo province. The department of Agriculture, Land Reform and Rural Development in the province is conducting a campaign to increase donkey production in the district, which has begun in three villages in Blouberg Municipality, namely Thorne, Archibalt, and Genau. The municipality is home to 175 753 people and 41 416 homes, accounting for 13.2 percent of the district’s population. The municipality is known for its warm winters, which are typically frost-free, and it’s extremely hot, often dry summers. The area receives approximately 455mm of annual rainfall, which mostly comes in the form of afternoon thunderstorms between November and March. The average high temperature is 26.02°C, while the average low temperature is 12.10°C.

### Sampling procedures

Blouberg Local Municipality and the three villages namely; Thorne, Archibalt, and Genau were purposively selected since the provincial department of Agriculture, Land Reform and Rural Development indicated that this local municipality has a higher population of donkeys. Snowball sampling was used to find the donkey farmers in the surveyed villages. The idea behind snowball sampling was that after the investigator interviewed the first donkey farmer(s), a chain of respondents was established in which the first respondents was to provide information for the next respondents, who then provided information for the third respondent, and so on. The study intended to interview a minimum of 30 donkey farmers, but only 21 were willing to participate in the study. Simple random sampling was employed to select 74 donkeys from the three villages.

### Experimental animals

The two donkey breeds identified in the study area were the Wild and Feral donkeys. These two breeds are distributed all over the world and are believed to live well in dry, rocky locations with temperatures surpassing 50°C [20].

### Animal management

The farmers who took part in the trial took exemplary care of their animals; the donkeys were housed in well-built kraals with 24-hour access to water and nutrient supplements like salt leaks. Injured or unwell donkeys were not employed in the study. A makeshift rope halter was employed to restrain the donkeys during data collecting following the procedure of Fraser [21].

### Data collection

**Fig 1:**
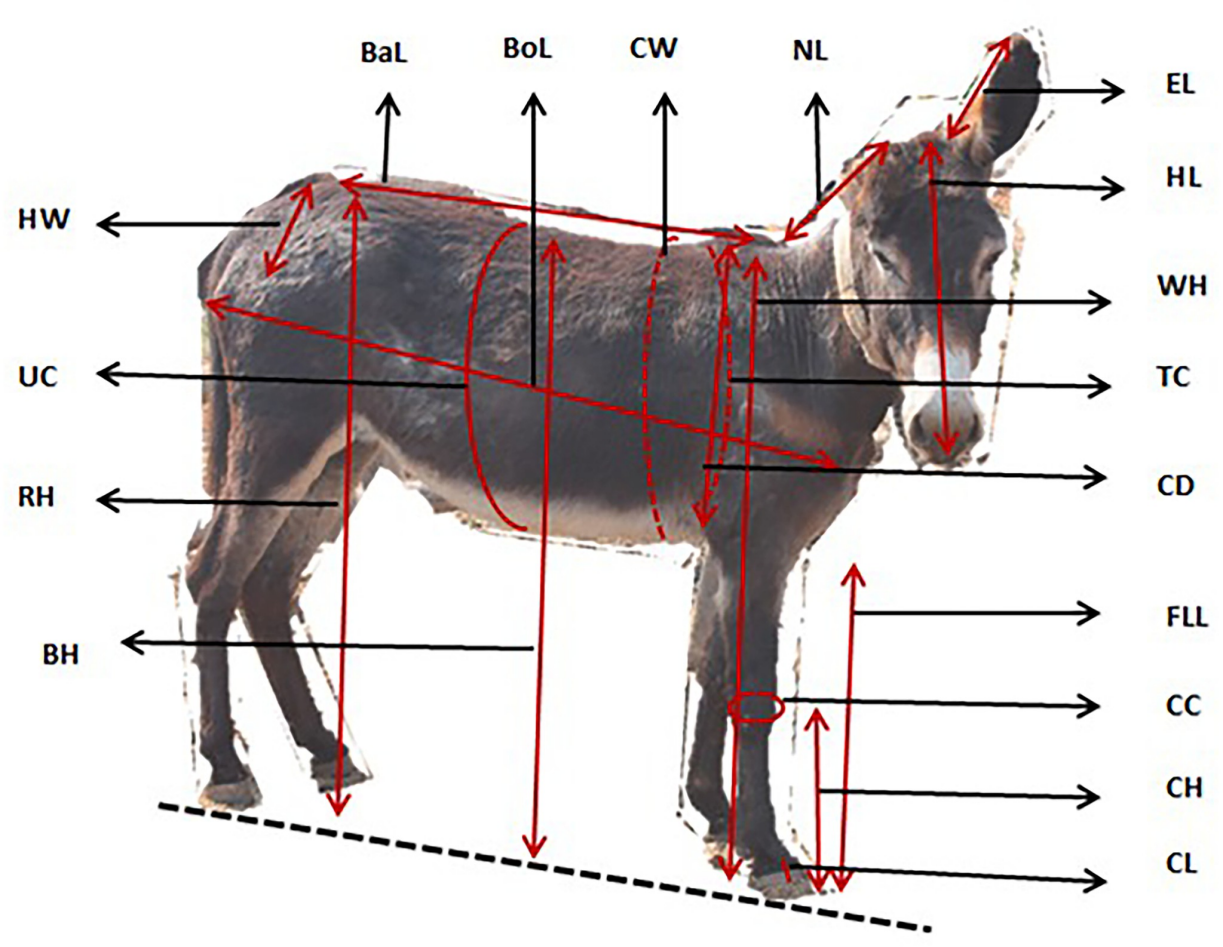
Different body measurements performed in donkeys.

Data was collected from individual donkey farmers through face-to-face interviews using the semi-structured questionnaire (S1 File), in three villages of the Blouberg Local Municipality in Limpopo Province. Questionnaires were created and translated into the local language then pre-tested to in 3 donkey farmers in each village to test whether the questions were clear and understandable to the farmers. Morphological traits were measured as shown in Fig 1 following the description of Ayad et al [15]. Briefly, Head Length (HL)—was measured from the space between the ears to the upper lip of the animal. Ear Length—measured from where the ear is joined to the head to the tip end of the ear. Neck Length—from where the head joins the neck towards the other end where the neck joins the body. Back length–measured from where the neck joins body to back rear back end. Thoracic Circumference–measured from the 4th lumber vertebra to the most proximal edge of the flank. Chest Width–measured from left end of chest to the right end. Body Length–measured as distance from point of shoulder to point of hip. Withers height–measured from the withers to the surface. Back height–from ground surface to the upper part of back. Front Leg Length–from surface to where leg joins body. Height at rump–from upper hip to the floor. Chest depth–measured from the whither to the upper part of front leg. Cannon Circumference–round measure of fore shank. Cannon height–measured from the cannon to the surface. Body weight was measured using weigh measuring tape (Rondo) calibrated in kilograms, using the hearth girth region just behind the shoulders in line with the methods explained by Vlaeva et al. [22].

### Statistical analysis

Data was analysed using Statistical Analysis Software version 9.4 [23]. The frequency procedure (PROC FREQ) was utilized to calculate frequencies and percentages. Chi-square test (χ2) was utilized to compare the categorical data among the surveyed villages. Mean procedure (PROC MEAN) was utilized to construct descriptive statistics of morphological features and Pearson’s correlation was used to determine the relationship between morphological traits. The following General Linear Model (PROC GLM) was employed to evaluate the obtained data:

Yij=u+Gi+eij

Where,

Yij is an observation of the morphological traits, u is the overall mean, Gi is the fixed effect of ith sex and eij is the residual error. Significance was observed at P<0.05 and P<0.01 for highly significance. Trait preferences were computed for the importance of each criterion and estimated by computing the index of ranking as discussed by Zwedu et al. [24]. Index = Sum (3 x rank1 + 2 x rank2 + 1 x rank3) for individual trait / Sum (3 x rank1 + 2 x rank2 + 3 x rank1) for overall traits.

## Results

### Socio-economic status of donkey farmers

Socio-economic characteristics of donkey farmers in the three selected villages are summarised in Table 1. The results revealed that there was no significant difference (P>0.05) between the three villages in all the socio-economic characteristics except educational level (P<0.05). The majority of donkey farmers surveyed were married male, average age of above 60 years (63–66) and most of them were illiterate in Thorp and Archibalt villages while were literate in Genau village.

**Table 1:** Socio-economic status of donkey farmers.

		Villages				
		Genau	Archibalt	Thorp		
Characteristic		N (%)	N (%)	N (%)	Chi-square	P-value
**Categorical variables**						
**Gender**						
Male		7 (87.50)	6 (100.00)	6 (85.71)		
Female		1 (12.50)	0 (0.00)	1 (14.29)	0.90	0.64^ns^
**Marital Status**						
Single		2 (25.00)	0 (0.00)	1 (14.29)		
Married		5 (62.50)	6 (100.00)	6 (85.71)		
Widow		1 (12.50)	0 (0.00)	0 (0.00)	3.75	0.44^ns^
**Level of Education**						
No Formal		0 (0.00)	1 (16.67)	5 (71.43)		
Primary school		2 (25.00)	4 (66.67)	1 (14.29)		
Secondary school		6 (75.00)	1 (16.67)	1 (14.29)	14.64	0.01*
**Money from Donkeys**						
Yes		5 (62.50)	6 (100.00)	4 (57.14)		
No		3 (37.50)	0 (0.00)	3 (42.86)	3.41	0.18^ns^
**Religion**						
Christian		6 (75.00)	4 (66.67)	6 (85.71)		
African Tradition		2 (25.00)	2 (33.33)	1 (14.29)	0.66	0.72^ns^
**Occupation**						
Public		7 (87.50)	6 (100.00)	4 (57.14)		
Private		1 (12.50)	0 (0.00)	0 (0.00)		
Pensioner		0 (0.00)	0 (0.00)	3 (42.86)	8.43	0.08^ns^
**Continuous variables**						
	**Mean±SEM**		**Mean±SEM**	**Mean±SEM**	**F-value**	**P-value**
Age	63.88±5.04		63.83±3.34	66.43±7.82	0.06	0.94^ns^
Household size	7.13±0.90		7.00±0.89	4.86±1.26	1.52	0.25^ns^
Years farming with donkeys	17.25±4.71		17.83±7.15	27.71±7.94	0.81	0.46^ns^
Number of donkeys	4.63±0.32		5.16±0.65	6.43±2.06	0.57	0.57^ns^

* Significant at P < 0.05

^ns^ = not significant, SE: Standard error of mean.

### Purpose of keeping donkeys

The purpose of keeping donkeys are presented in Table 2. The findings showed that there was no significant difference (P>0.05) in purpose of keeping donkeys among villages. The majority of farmers kept donkeys for drought power and cart pulling in all the surveyed villages.

**Table 2:** Purpose of keeping donkeys.

Purpose	Villages			Chi-square	P-value
	Genau N (%)	Archibalt N (%)	Thorp N (%)		
Cart pulling	3 (37.50)	0 (0.00)	1 (14.29)		
Drought power and cart pulling	5 (62.50)	5 (83.33)	5 (71.42)		
Social status, drought power and cart pulling	0 (0.00)	1 (16.67)	0 (0.00)		
Milk production, social status, drought power and cart pulling	0 (0.00)	0 (0.00)	1 (14.29)	7.36	0.29^ns^

^ns^ = not significant.

### Donkey breeds kept and breeding practices

Types of donkey breeds kept and breeding practices conducted by the donkey famers in the three selected villages are presented in Table 3. The results indicated that there was no statistical difference (P>0.05) between the villages on donkey breeds kept. Controlled mating, practice culling, reasons for culling and breeding seasons had no significant different (P>0.05) among the villages. The results showed a significant difference (P<0.05) in inbreeding knowledge by the farmers among the three villages. Most of the donkey farmers in surveyed villages had uncontrolled mating during the spring season (September, October and November).

**Table 3:** Donkey breeds kept and breeding practices of donkey farmers.

Characteristic	Villages			Chi-square	P-value
	Genau N (%)	Archibalt N (%)	Thorp N (%)		
**Donkey breeds kept**					
Wild	6 (75.00)	2 (33.33)	5 (71.43)		
Feral	2 (25.00)	4 (66.67)	2 (28.57)	2.93	0.23^ns^
**Controlled mating**					
Yes	2 (25.00)	1 (16.67)	3 (42.86)		
No	6 (75.00)	5 (83.33)	4 (57.14)	1.17	0.56^ns^
**Inbreeding known**					
Yes	8 (100.00)	6 (100.00)	3 (42.86)		
No	0 (0.00)	0 (0.00)	4 (57.14)	9.88	0.01*
**Culling practiced**					
Yes	1 (12.50)	2 (33.33)	5 (71.43)		
No	7 (87.50)	4 (66.67)	2 (28.57)	5.58	0.06^ns^
**Reasons for culling**					
Old age	5 (62.50)	2 (33.33)	4 (57.14)		
Low production	3 (37.50)	4 (66.67)	3 (42.86)	1.26	0.53^ns^
**Mating season**					
Spring	6 (75.00)	6 (100.00)	7 (100.00)		
Autumn	2 (25.00)	0 (0.00)	0 (0.00)	3.59	0.17^ns^

* = significant at P <0.05

^ns^ = not significant.

### Selection criterions of donkey farmers

**Fig 2:**
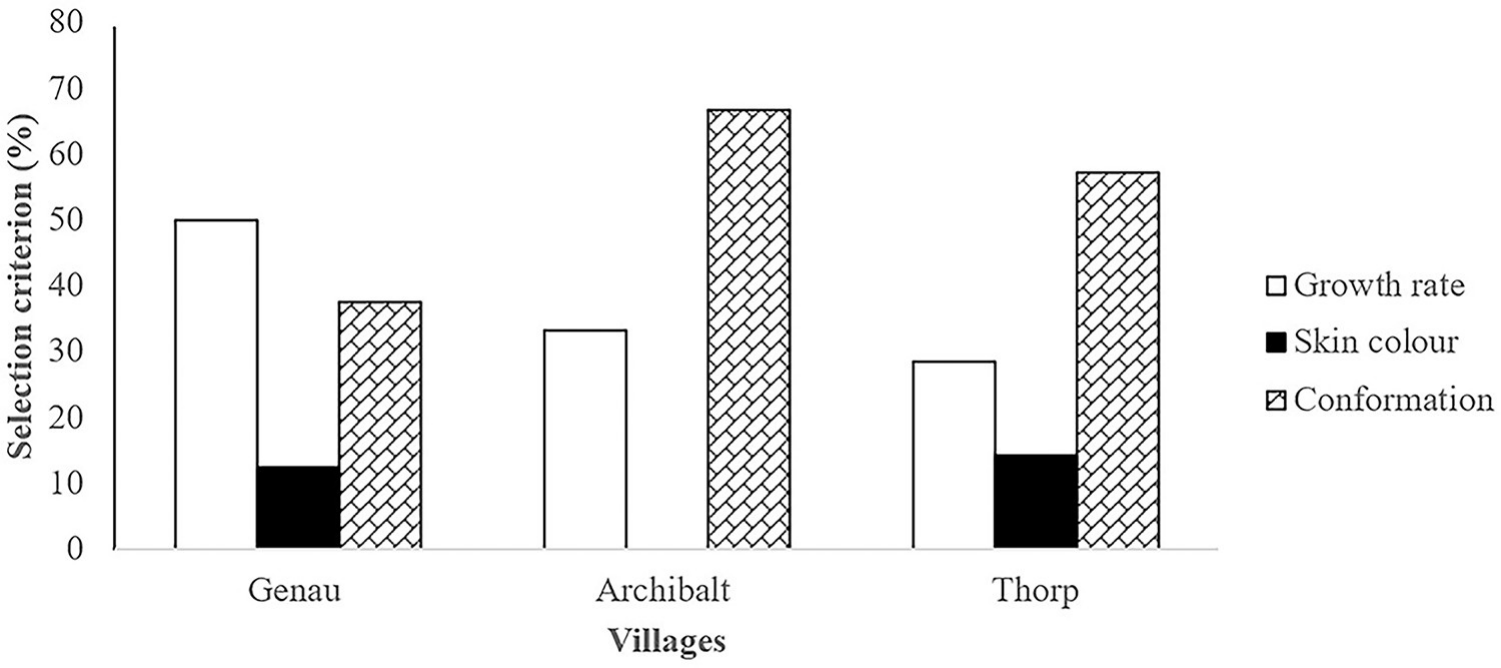
Selection criterion used by donkey farmers.

The selection criterions used by the donkey farmers in the three villages are summarized in Fig 2. The results indicated that the donkey farmers used the same selection methods in all three villages (P>0.05). Growth rate was the most used selection criteria in Genau village while body confirmation was the most used selection criteria in Archibalt and Thorp villages.

### Ranks and indices for trait preference in male donkeys

Ranks and indices for trait preference in male donkeys are presented in Table 4. Index was performed for calculating the significance of the traits for all the surveyed villages. The results revealed that overall, body size (0.39), growth rate (0.19), animal performance (0.14) and mating ability (0.11) were identified as the important traits for the selection of male donkeys.

**Table 4:** Ranks and indices for trait preference in male donkeys.

Traits	Villages												Overall Index
	Genau				Archibalt				Thorp				
	R1	R2	R3	Index	R1	R2	R3	Index	R1	R2	R3	Index	
Mating Ability	0	0	1	0.03	0	1	1	0.10	3	1	2	0.20	0.11
Body size	3	1	0	0.37	4	1	1	0.50	2	6	1	0.29	0.39
Ear Size	1	0	0	0.10	0	0	0	0.00	1	1	2	0.11	0.07
Coat colour	0	0	1	0.03	0	0	1	0.03	1	0	1	0.06	0.04
Growth rate	0	3	0	0.20	0	2	0	0.13	2	3	3	0.23	0.19
Performance	1	0	3	0.20	1	0	2	0.17	1	0	1	0.06	0.14
Temperament	0	1	0	0.07	0	1	0	0.07	1	0	1	0.06	0.07

R1 –R3 = Rank 1 to Rank 3.

### Ranks and indices for trait preference in male donkeys

Table 5 summarises the ranks and indices for trait preference in female donkeys of all the three villages. The findings indicated that overall, body size (0.26), twinning ability (0.23), mothering ability (0.15) and foaling ability (0.10).

**Table 5:** Ranks and indices for trait preference in female donkeys.

Traits	Villages												Overall Index
	Genau				Archibalt				Thorp				
	R1	R2	R3	Index	R1	R2	R3	Index	R1	R2	R3	Index	
Twinning ability	3	0	0	0.30	1	1	0	0.17	2	3	3	0.23	0.23
Body size	1	0	0	0.10	3	1	1	0.40	6	0	1	0.29	0.26
Mothering ability	1	3	0	0.30	0	1	1	0.10	0	1	1	0.05	0.15
Coat colour	0	0	0	0.00	0	1	0	0.07	0	2	1	0.08	0.05
Age at first foaling	0	0	2	0.07	0	0	1	0.03	0	1	2	0.06	0.05
Foaling ability	0	1	2	0.13	1	0	2	0.17	1	1	1	0.09	0.10
Performance	0	1	0	0.07	0	0	0	0.00	1	2	0	0.11	0.06
Temperament	0	0	1	0.03	0	1	0	0.07	1	1	2	0.11	0.07

R1 –R3 = Rank 1 to Rank 3.

### Donkey variation in colour

**Fig 3:**
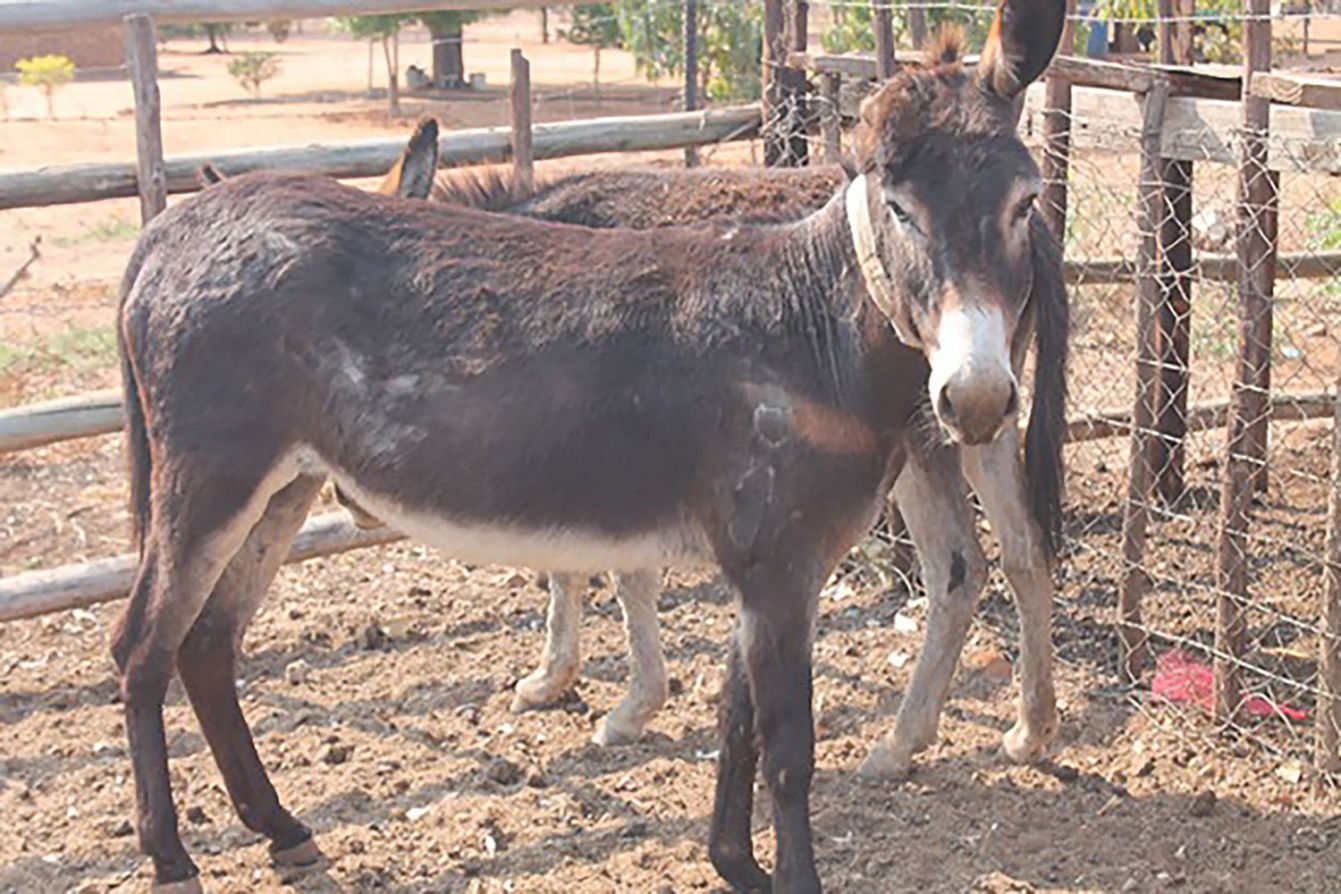
Dark brown donkey.

**Fig 4:**
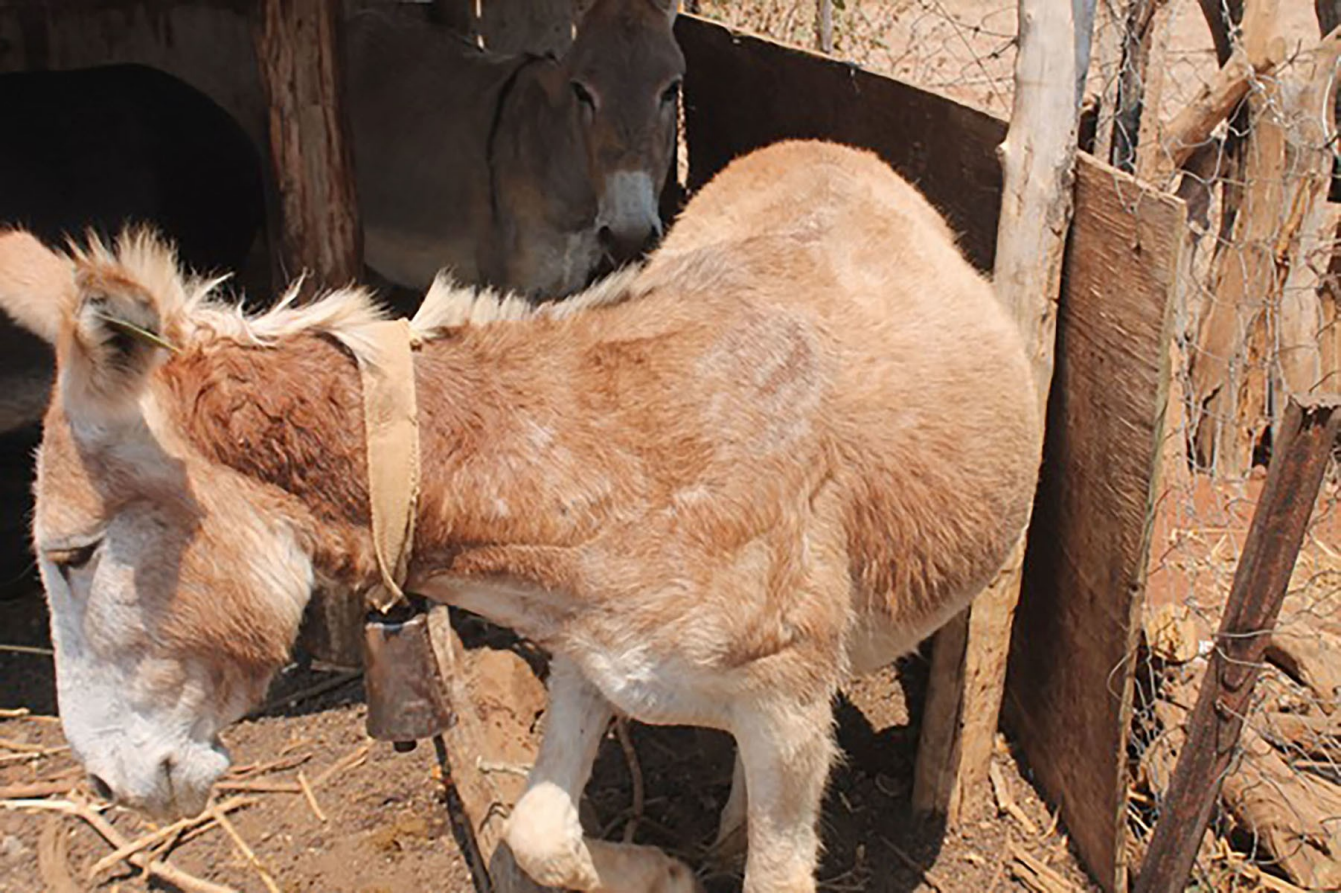
Light brown donkey.

**Fig 5:**
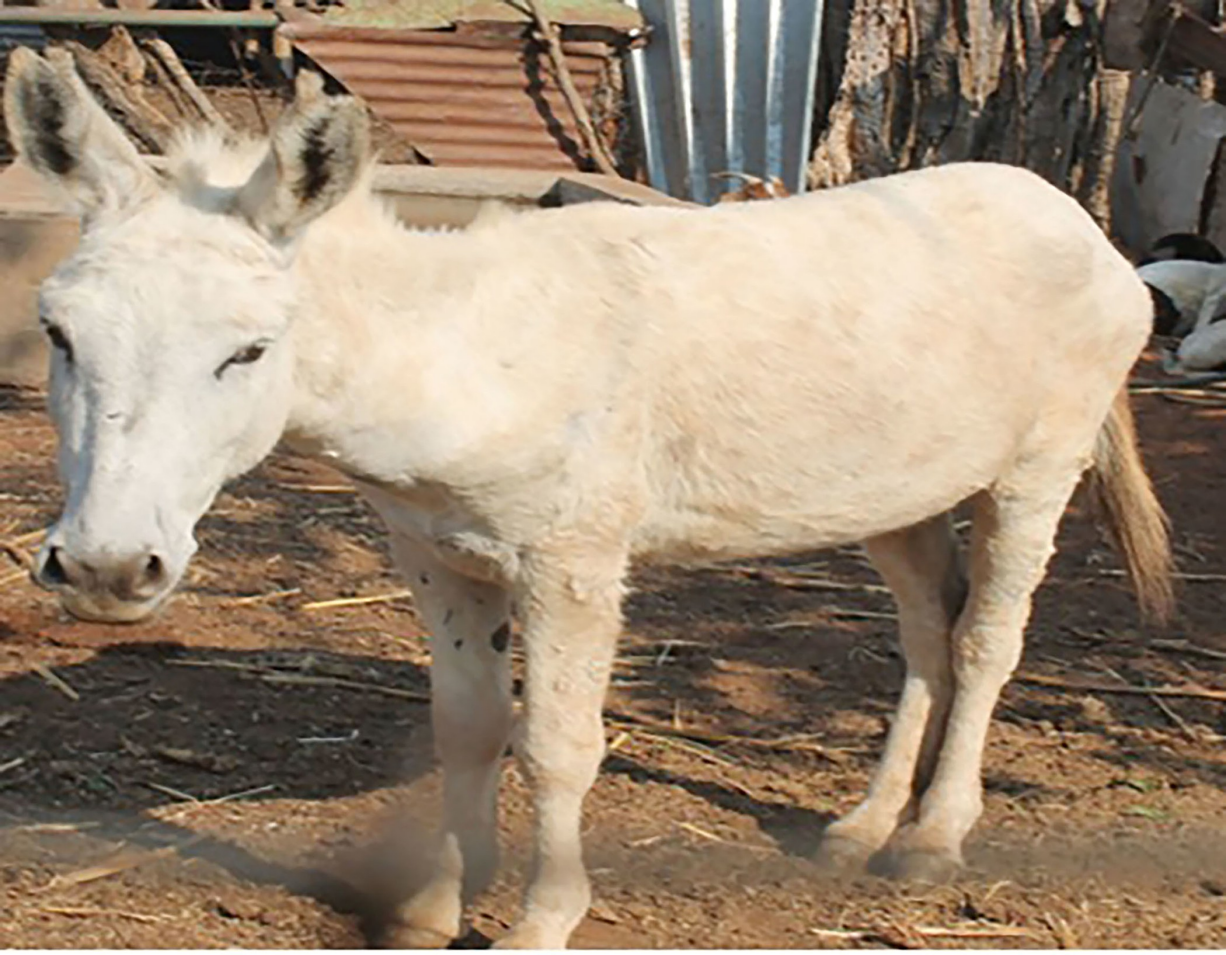
White donkey.

**Fig 6:**
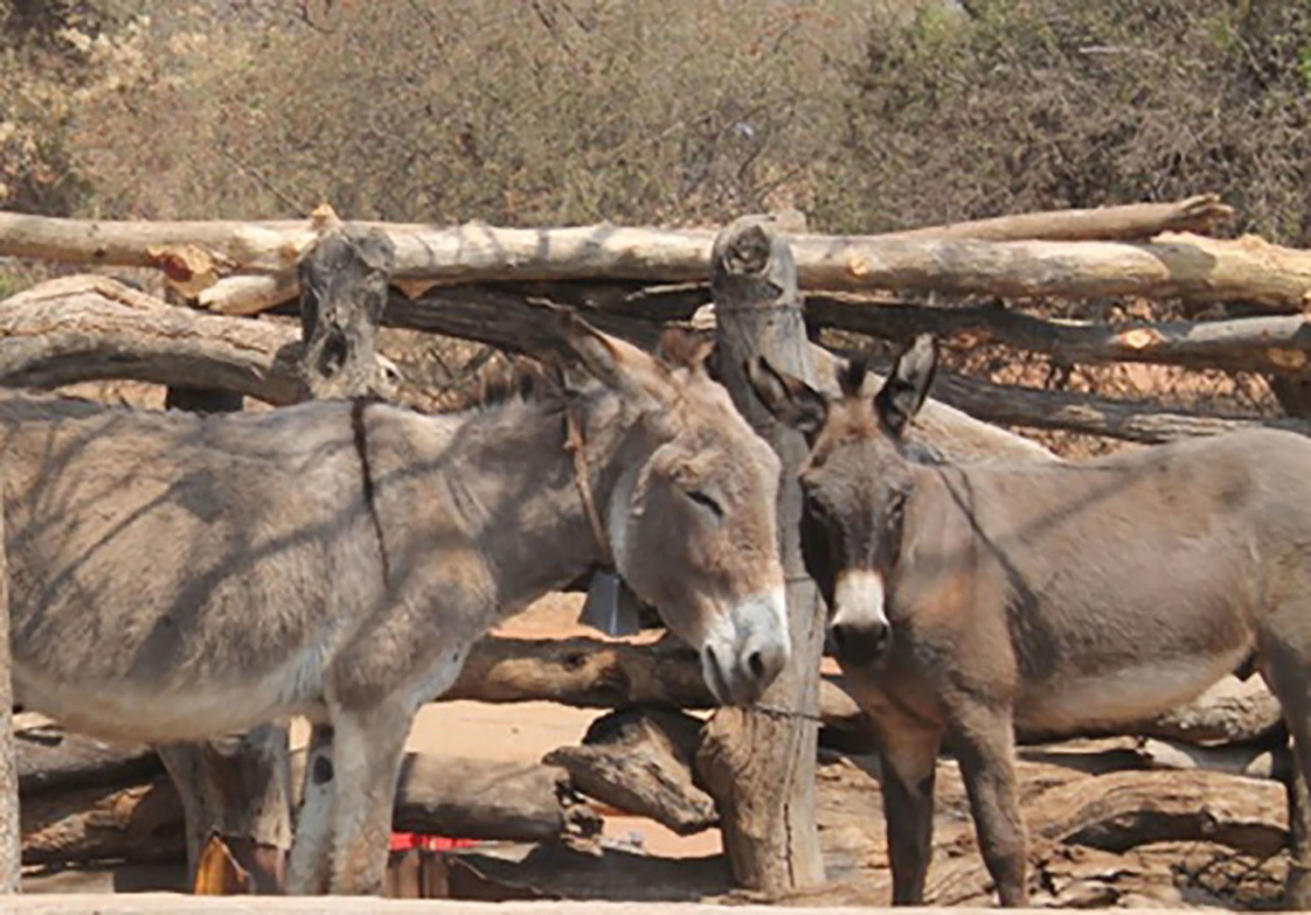
Grey donkey.

**Fig 7:**
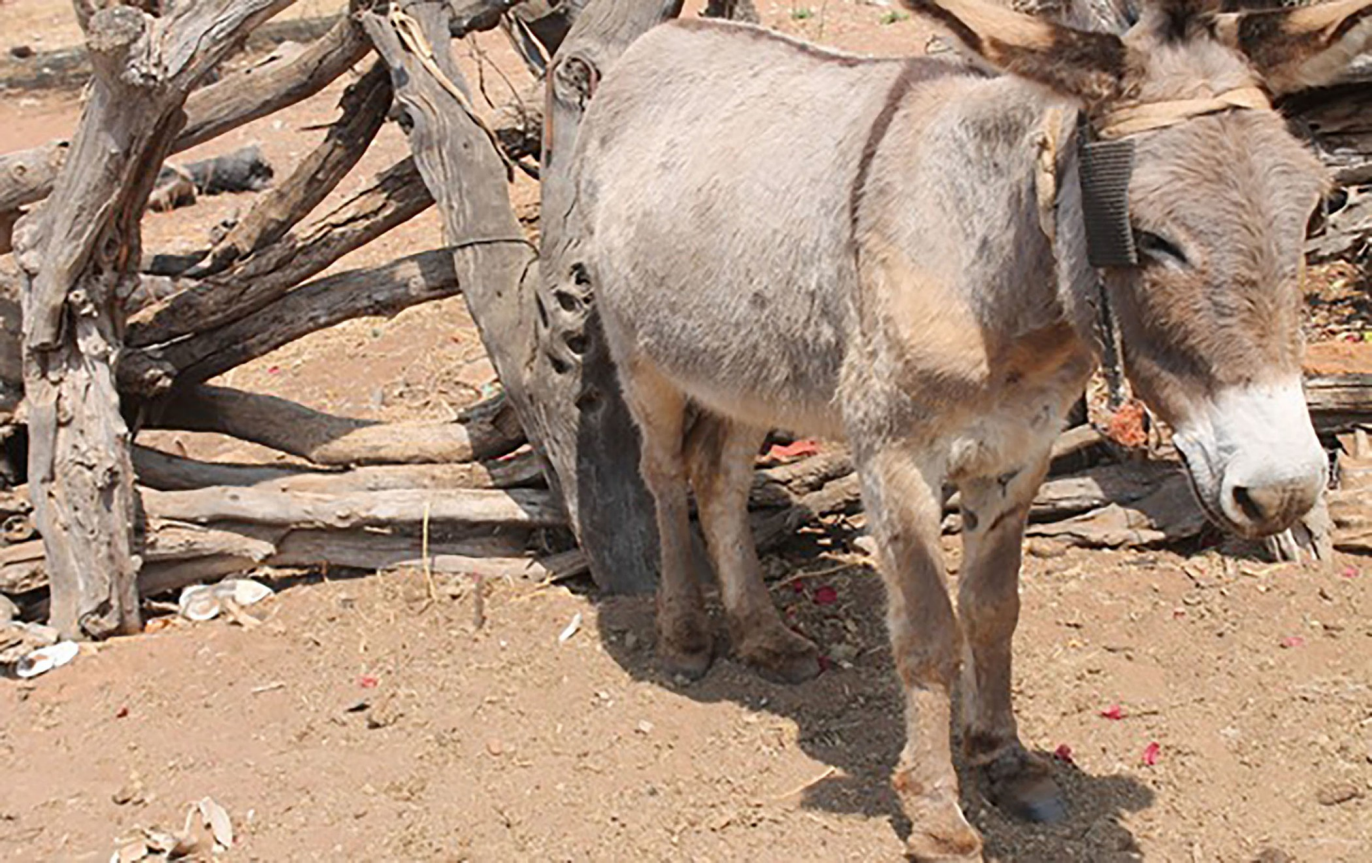
Chuck coal donkey.

Donkey colour variations were noticed in the investigation on three separate villages, as shown in Table 6. The donkey colours in the three communities were not statistically substantially different (P>0.05), and the same colours existed in each hamlet. In the current study, the donkeys’ colours were mostly dark brown (Fig 3), light brown (Fig 4), white (Fig 5), grey (Fig 6), and chuck coal (Fig 7).

**Table 6:** Donkey colour variations.

Coat colour	Villages			Chi-square	P-value
	Genau N (%)	Archibalt N (%)	Thorp N (%)		
Dark brown	16 (39.02)	5 (27.78)	5 (33.33)		
Light brown	7 (17.07)	3 (16.67)	3 (20.00)		
White	4 (9.76)	3 (16.67)	0 (0.00)		
Grey	13 (31.71)	7 (38.89)	7 (46.67)		
Chuck coal	1 (2.44)	0 (0.00)	0 (0.00)	4.46	0.81^ns^

^ns^ = not significant.

### Effect of village on the morphological features and body weight of the donkeys

The results of the effect of village on the morphological features and body weight of the donkeys are shown in Table 7. The findings indicated that there was significant different (P<0.05) observed on BaL, BH and FLL between the three villages. The results also revealed that there was a highly significant difference (P<0.01) observed in HW among surveyed villages. Longer BaL was observed in donkeys from Archibalt (80.39±084) and Thorp (78.93±1.25) villages. The current results indicated that donkeys from Thorp village had a higher HW (48.80±5.82) as compared to other villages.

**Table 7:** Effect of village on morphological traits and body weight of donkeys.

	Villages			
	Genau	Archibalt	Thorp	
Traits	Mean±SEM	Mean±SEM	Mean±SEM	P-value
HL (cm)	48.61±0.88	50.29±0.60	50.27±1.01	0.28^ns^
EL (cm)	25.94±0.90	25.09±0.80	25.73±0.69	0.77^ns^
NL (cm)	47.30±1.04	48.12±0.96	50.47±1.55	0.26^ns^
CW (cm)	121.60±1.07	124.44±0.93	124.47±1.16	0.15^ns^
BaL (cm)	74.90±1.71^b^	80.39±0.84^a^	78.93±1.25^a^	0.01*
BoL (cm)	118.00±1.29	119.90±1.28	121.07±3.33	0.60^ns^
HW (cm)	34.83±1.44^b^	40.37±0.86^b^	48.80±5.82^a^	0.003**
UC (cm)	75.94±1.67	79.54±0.98	76.93±2.80	0.20^ns^
BH (cm)	115.61±1.06^ab^	118.56±0.94^a^	113.73±2.54^b^	0.04*
HR (cm)	118.78±1.28	120.88±0.98	121.87±1.05	0.27^ns^
TC (cm)	25.72±1.11	27.66±0.67	29.20±0.69	0.06^ns^
CD (cm)	43.94±0.98	44.32±0.85	45.40±0.69	0.65^ns^
WH (cm)	111.39±1.07	113.07±0.83	113.13±1.31	0.47^ns^
FLL (cm)	74.33±1.1^ab^	71.54±1.2^b^	77.60±1.06^a^	0.01*
CC (cm)	23.05±0.76	23.51±0.25	24.13±0.45	0.35^ns^
CL (cm)	34.39±1.36	34.46±0.60	35.40±0.56	0.73^ns^
CH (cm)	6.67±0.26	6.80±0.20	6.73±0.39	0.93^ns^
BW (kg)	105.83±2.50	108.93±1.75	109.27±2.68	0.56^ns^

* = significant at P < 0.05

**Highly significant (P<0.01)

^ns^ = not significant

a, b: Means in the same row with different superscripts are significantly (P<0.05), SEM—Standard error of mean; HL—Head length; EL—Ear length; NL—Neck length; CW—Chest width; Bal—Back length; BoL—Body length; HW—Hip width; UC—Umbilical circumference; BH—Back height; HR—Height at the rump; TC—Thoracic circumference; CD—Chest depth; WH—Withers Height; FLL—Front leg length; CC—Cannon circumference; CL -Cannon length; CH—Cannon height; BW–Body weight.

### Phenotypic correlation between measured traits

Phenotypic correlation of 18 traits measured of female and male donkeys that existed in three villages are presented in Table 8. BW in male donkeys showed not to be significantly correlated (P>0.05) with HL, EL, NL, BaL, HW, FLL, CC, CH, CL but was positively statistically correlated (P<0.05) with BoL, UC, TC and CD. In female donkey, the findings indicated that BW had a positively high significant correlation (P<0.01) with CW, BH, HR and WH but not statistically correlated (P>0.05) with NL, BaL, TC and CL.

**Table 8:** Phenotypic correlation between measured traits, male above diagonal and female below diagonal.

Traits	HL	EL	NL	CW	BaL	BoL	HW	UC	BH	HR	TC	CD	WH	FLL	CC	CL	CH	Bwe
**HL**		-0.09^ns^	-0.28^ns^	0.20^ns^	0.27^ns^	-0.12^ns^	0.20^ns^	0.01^ns^	0.14^ns^	0.28^ns^	0.28^ns^	-0.09^ns^	0.31*	0.13^ns^	0.37*	0.19^ns^	0.31^ns^	0.19^ns^
**EL**	0.33^ns^		-0.18^ns^	0.22^ns^	0.17^ns^	0.09^ns^	0.17^ns^	0.04^ns^	-0.09^ns^	0.19^ns^	0.03^ns^	0.05^ns^	0.16^ns^	0.05^ns^	-0.12^ns^	-0.00^ns^	0.03^ns^	0.15^ns^
**NL**	0.17^ns^	0.05^ns^		0.20^ns^	-0.36*	0.33*	0.09	0.22^ns^	0.04^ns^	0.23^ns^	0.50**	-0.04^ns^	-0.03^ns^	0.44**	0.16^ns^	0.08^ns^	0.08^ns^	0.19^ns^
**CW**	0.47**	0.38*	0.19^ns^		0.52**	0.35*	0.09^ns^	0.33*	0.45**	0.63**	0.30^ns^	0.38*	0.61**	0.25^ns^	0.35*	0.17^ns^	0.11^ns^	0.94**
**BaL**	0.33^ns^	0.07^ns^	-0.16^ns^	0.49**		0.88^ns^	0.89^ns^	0.97^ns^	0.04^ns^	0.11^ns^	0.47^ns^	0.03^ns^	0.00^ns^	0.59^ns^	0.54^ns^	0.46^ns^	0.64^ns^	0.00^ns^
**BoL**	0.41*	0.12^ns^	0.59**	0.48**	0.04^ns^		-0.40*	0.59**	0.51**	0.19^ns^	0.37*	-0.01^ns^	0.15^ns^	0.21^ns^	0.12^ns^	0.04^ns^	0.22^ns^	0.34*
**HW**	0.30^ns^	0.10^ns^	0.21^ns^	0.51**	0.40*	0.36*		-0.41**	-0.56**	0.29^ns^	0.37*	-0.03^ns^	0.07^ns^	0.22^ns^	0.20^ns^	0.10^ns^	0.04^ns^	0.14^ns^
**UC**	0.53**	0.37*	0.24^ns^	0.43*	0.18^ns^	0.35*	0.13^ns^		0.59**	0.09^ns^	0.35*	0.07^ns^	0.16^ns^	-0.06^ns^	-0.01^ns^	-0.41**	-0.03^ns^	0.35*
**BH**	0.52**	0.22^ns^	0.06^ns^	0.79**	0.50**	0.33^ns^	0.41*	0.62**		0.36*	0.09^ns^	0.23^ns^	0.44**	-0.07^ns^	0.21^ns^	-0.04^ns^	0.11^ns^	0.41**
**HR**	0.45**	0.25^ns^	0.20^ns^	0.79**	0.30^ns^	0.57**	0.54**	0.44**	0.79**		0.47**	0.25^ns^	0.56**	0.49**	0.41**	0.43**	0.16^ns^	0.60**
TC	0.30^ns^	-0.06^ns^	0.45**	0.39*	0.04^ns^	0.39*	0.27^ns^	0.15^ns^	0.33^ns^	0.45**		-0.20^ns^	0.18^ns^	0.47**	0.36*	0.07^ns^	0.25^ns^	0.33*
CD	0.53**	0.34*	0.20^ns^	0.82**	0.47**	0.53**	0.43*	0.45**	0.84**	0.81**	0.39*		0.39*	-0.08^ns^	0.19^ns^	-0.06^ns^	-0.14^ns^	0.31*
WH	0.14^ns^	0.04^ns^	0.57**	0.26^ns^	-0.29^ns^	0.59**	0.10^ns^	0.18^ns^	0.17^ns^	0.35*	0.44**	0.03^ns^		0.29^ns^	0.30^ns^	0.21^ns^	-0.07^ns^	0.58**
FLL	0.17^ns^	0.15^ns^	0.18^ns^	0.37*	-0.05^ns^	0.30^ns^	0.14^ns^	0.28^ns^	0.33^ns^	0.44**	0.70**	0.28^ns^	0.39*		0.12^ns^	0.56**	0.14^ns^	0.27^ns^
CC	0.30^ns^	0.16^ns^	0.45**	0.44**	-0.04^ns^	0.68**	0.39*	0.34*	0.36*	0.59**	0.06^ns^	-0.24^ns^	0.46**	0.30^ns^		0.09^ns^	0.19^ns^	0.30^ns^
CL	0.30^ns^	-0.15^ns^	0.17^ns^	0.10^ns^	-0.24^ns^	0.41*	0.13^ns^	0.22^ns^	0.25^ns^	0.36*	0.27^ns^	0.18^ns^	0.27^ns^	0.30^ns^	-0.15^ns^		0.55**	0.18^ns^
CH	0.29^ns^	-0.17^ns^	0.19^ns^	0.04^ns^	-0.34^ns^	0.44*	0.16^ns^	0.42^ns^	0.23^ns^	0.38*	0.26^ns^	0.15^ns^	0.24^ns^	0.29^ns^	-0.14^ns^	0.29^ns^		0.09^ns^
Bwe	0.43*	0.37*	0.17^ns^	0.75**	0.27^ns^	0.36*	0.34*	0.44*	0.65**	0.64**	0.14^ns^	0.10^ns^	0.67**	0.43*	0.37*	0.17^ns^	0.75**	

* = significant at P < 0.05, ** = high significant at P < 0.01, ^ns^ = not significant; HL—Head length; EL—Ear length; NL—Neck length; CW—Chest width; Bal—Back length; BoL—Body length; HW–Hip; * = significant at P < 0.05, ** = high significant at P < 0.01, ^ns^ = not significant; HL—Head length; EL—Ear length; NL—Neck length; CW—Chest width; Bal—Back length; BoL—Body length; HW—Hip width; UC—Umbilical circumference; BH—Back height; HR—Height at the rump; TC—Thoracic circumference; CD—Chest depth; WH—Withers Height; FLL—Front leg length; CC—Cannon circumference; CL -Cannon length; CH—Cannon height and BW–Body Weight.

## Discussion

A better understanding of livestock farmers’ production objectives and breeding practices, and also livestock morphological characterization is fundamental to design and implement breeding programme at the community level [25,26]. Socio-economic characteristics of all the participated donkey farmers in the surveyed villages of Blouberg Local Municipality in Limpopo province, South Africa were documented. The findings discovered that there was no significant different observed on all studied socio-economic characteristics of donkey farmers except on education level among surveyed villages. Our findings are in agreement with the study of Swai and Bwanga [27], who discovered that donkey farmers’ educational levels ranged from no formal to secondary school education in northern Tanzania. The current study suggests that donkey farmers in the studied villages have just a basic education that does not extend beyond secondary school. Average age of donkey farmers of the three villages was found ranging 63.88 to 66.43. According to Shuiep [28] the average age of donkey farmers ranges around 32.8, with farmers having only one donkey, which was utilized to earn income or to be ridden for transportation. In this survey, no young farmers owned donkeys; most farmers were in their retirement years (above sixty years). A donkey is an animal that is the most efficient agricultural power unit [29] and are kept for a variety of reasons, including milk, meat, production labour and recreation [2]. The current study also investigated the purpose of keeping donkeys in all the three villages. The results discovered that donkeys were mostly used to assist farmers with drought power, cart pulling, social status, and donkey product consumption, but primarily for drought power and cart pulling. Donkey farmers in the study conducted by Hassan et al. [5] focused on employing donkeys for income-generating activities rather than on drought power. The wild and the feral donkey were the breeds kept by farmers, which agreed with the conclusions of Kimura et al. [30] indicated that at least three different varieties of wild asses are found in Africa. Breeding practices of the donkey farmers were studied in all the three villages. According to Tyasi et al. [6] livestock farmers approach is important for the development of livestock improvement programme. The findings revealed that, while donkey farmers were aware of ideas such as inbreeding, there was no breeding programme in place to aid in the preservation of particular donkey genetic features required for reproduction and assisting in genetic variability among the villages. Similar findings were observed by Hassan et al. [5] where farmers were aware of inbreeding but did little to nothing about it. A study conducted by Nigussie et al. [31] observed similar results to this study, where mating of animals in a communal production system was not controlled and animals mated randomly. Trait preferences by farmers is a powerful tool for livestock farmers for ranking their animals [32]. The current study found that body size, growth rate and mothering skill were among the most highly valued features in the selection of donkeys to be the parents of the future generation, however coat colour was not. In use, the size of the donkey has a significant impact on the task that it will execute. Abebe et al. [25] discovered that physical visual features such as body size and coat colour were the most important in the selection of breeding animals for small scale farmers. Misganaw et al. [12] discovered that community farmers ranked drought power and animal outputs in the form of milk highly. In this study, performance was ranked as one of the favoured features, although it did not score highly, and none of the farmers were interested in the animal outputs such as milk or meat; instead, the farmers were interested in the animals’ capacity to walk. Across all the three surveyed villages, body size growth rate, animal performance and mating ability were recognized as the important traits for the selection of male donkeys while body size, twinning ability, mothering ability and foaling ability for female donkeys were considered as the most preferred traits. Abebe et al. [25] discovered that physical visual features such as body size and coat colour were the most important in the selection of breeding animals for small scale farmers. Trait preference findings of the current study suggest that body size is the most preferred trait for both male and female donkeys, this is because body size helps the farmers to pull the cart as the primary purpose of the donkey farmers to keep them. In the current study, donkeys’ colours were mostly brown (dark and light), white, grey and chuck coal. These colours matched the findings of Bunevski et al. [33] who discovered that the colours of Macedonian donkeys were brown, black, and grey. This work study region was topographically similar to the Macedonian donkey study site however; the current study site was hilly with extreme hot temperatures. To fully aid decision making on breeding programme construction, a thorough understanding of breed traits is required [34]. The majority of the morphological features measured in the study did not differ between the three villages. There was a difference in front leg length, body height, height at withers and back length; the acquired results differed with modest similarities from a study conducted by Sargentini et al. [17], who discovered a number of variances between morphological traits identified in different regions. The current study used villages that were quite close to each other and had the same climatic circumstances; therefore, there was no variation in the measured attributes because the animals were all from the same location. Nengomasha et al. [18] stated unequivocally that there is very little physical variation in African donkeys; however, a study conducted in Kenya by Gichure et al. [35] discovered the higher body weight, body length, height at whither, and heart girth. Our results had the agreement with higher body length, wither height, and chest width. Correlation results indicated that some of the morphological traits were correlated. Dissimilar findings were observed in the study of Gurcan et al. [19] who discovered that all the morphometric traits were highly correlated for donkey populations reared in Turkey. The current study suggests that the morphological characterization of donkeys need to be considered in designing and implementing CBBPs in the three surveyed villages.

## Conclusion

The majority of donkey farmers in the surveyed villages were older than 60 years and kept their donkeys for drought power and cart pulling. Wild and Feral were the only two donkey breeds found in the studied villages with dominant dark brown, light brown, white and grey coat colour and the mating was not controlled but the donkey farmers had the knowledge of inbreeding. Donkey famers have shown their highest preference for body size of male donkeys followed by growth rate, animal performance and mating ability while their highest preference in female donkeys have shown for body size followed by twinning ability, mothering ability and foaling ability. Variations were observed in back length, back height, front leg length and hip width as morphological characteristics of donkeys among the three surveyed villages and some of the morphological traits were correlated with body weight. Therefore, breeding practices, trait preferences and morphological traits discovered in the current study need to be considered in designing and implementing of community-based breeding programmes in the studied villages of Blouberg Local Municipality, Limpopo province of South Africa.

## Supporting information

S1 File The questionnaire used in the survey. The questionnaire developed to collect socio-economic characteristics and donkey breeding practices data.
(PDF)

S2 File The consent form used in the survey. The documented consent form was developed to be signed by each donkey farmer before commence data collection.
(PDF)

S3 File Raw data for socio-economic characteristics of donkey farmers. It is an Excel file with all the variables explained fully in column M of the sheet.
(XLSX)

S4 File SAS Programming for socio-economic characteristics of donkey farmers. It is an SAS file with all the syntax used for statistical analysis of socio-economic characteristics of donkey farmers’ data.
(SAS)

S5 File Raw data for breeding practices. It is an Excel file with variables fully explained in column I of the sheet.
(XLSX)

S6 File SAS Programming for breeding practices. It is an SAS file with all the syntax used for statistical analysis of breeding practices of donkey farmers’ data.
(SAS)

S7 File Raw data for morphological characterization of donkeys. It is an Excel file for all morphometric traits fully explained in raw 76 of the sheet.
(XLSX)

S8 File SAS Programming for data analysis of morphological characterization of donkeys. It is an SAS file with all the syntax used for statistical analysis.
(SAS)
