# Supplementary material for: Evaluation of breeding practices and morphological characterization of donkeys in Blouberg Local Municipality, Limpopo province: Implication for the design of community-based breeding programme
Source: PLoS One. 2022 Dec 14;17(12):e0278400. doi: 10.1371/journal.pone.0278400 (PMC9750015; doi:10.1371/journal.pone.0278400)
Supplement: S1 File — The questionnaire developed to collect socio-economic characteristics and donkey breeding practices data. (PDF) [file pone.0278400.s001.pdf]

S1 File. The questionnaire used in the survey

The survey was conducted in three selected villages (Thorne, Archibalt, and Genau) of Blouberg Municipality, Limpopo Province, South Africa.

Department of Agricultural Economics and Animal Production,

University of Limpopo,

Private Bag X1106, Sovenga 0727,

South Africa

Questionnaire: Evaluation of breeding practices and morphological characterization of donkeys in Blouberg Local Municipality, Limpopo province: Implication for the design of community-based breeding programme

The main aim of this study is to investigate donkey farmers breeding practices and donkey's morphological characterisation in the Blouberg local Municipality, Limpopo South Africa.

**QUESTIONNAIRE NUMBER**	

**NAME OF RESPONDENT**	
**CONTACT DETAILS**	

**NAME OF THE VILLAGE**<br>**. SECTION A: SOCIO-ECONOMIC QUESTIONS**	

1. SECTION A: SOCIO-ECONOMIC CHARACTERISTICS OF RESPONDENTS

1.1 Gender of household's head

1. Male		2. Female	

1.2 Age of the respondents…………………………………………………….

1.3 Marital status

1. Single		2. Married		3. Widow		4. Divorced	

1.4 Household size…………………………………………………………………..

1.5 Level of education

1. No formal Education		2. Primary		3. Secondary		4. Tertiary	

1.6 How many donkeys do you have?……………………………………………....

1.7 How long have you farmed with donkeys?...

1.8 Do you make money from donkeys?

1. Yes		2. No	

1.9 What is your religion?

1. Christian		2. Muslim		3. African tradition		4. Other	

If other please specify ……………………………………………

1.10 Occupation

1. Public		2. Private		3. Pensioner		4. Other	

If other please specify ……………………………………..

2. SECTION B: BREEDING PRACTICES

2.1 Donkey breed type

Type of breed	1. Wild Donkey		2. F eral donkey		3. Istrain Donkey		4. Other

If others, please specify ………………………………………………………………

2.2 What is the purpose of keeping donkeys?

1. Meat consumption		2. Milk production		3. Social status		4. Drought power		5. Cart pulling	

2.3 Do you control mating of donkeys?

1. Yes		2. No	

2.4 Do you know the inbreeding concept?

1. Yes		2. No	

2.5 Do you practice culling?

1. Yes		2. No	

2.6 Reasons for culling

1. Old age		2. Low production		3. Others	

2.7 What is your breeding season?

1. Autumn		2. Spring	

3. SECTION C: Selection criterion

3.1 Which criteria do you use to select your male and female donkey for breeding?

1. Growth rate		2. Skin colour		3. Conformatio		4. Others	

If others, specify…………………………………………………………………………

3.2 Traits preferred in male donkeys

Trait	Response (Rank)
Mating ability	
Body size	
Ear size	
Coat colour	
Growth rate	
Temperament	
Other (specify)	

3.3 Traits preferred in female donkeys

Traits	Response (Rank)
Twinning ability	
Body size	
Mothering ability	
Temperament	
Age at 1st foaling	
Coat colour	

Foaling ability	
Other (specify)	
