# Supplementary material for: Evaluation of breeding practices and morphological characterization of donkeys in Blouberg Local Municipality, Limpopo province: Implication for the design of community-based breeding programme
Source: PLoS One. 2022 Dec 14;17(12):e0278400. doi: 10.1371/journal.pone.0278400 (PMC9750015; doi:10.1371/journal.pone.0278400)
Supplement: S2 File — The documented consent form was developed to be signed by each donkey farmer before commence data collection. (PDF) [file pone.0278400.s002.pdf]

S2 File. Consent form

University of Limpopo

School of Agriculture and Environmental sciences

Department of Agricultural Economics and Animal Production

Private Bag X1106, Sovenga, 0727, South Africa

Email: maswanamasixole@gmail.com

INFORMED CONSENT FORM FOR PARTICIPATING IN A RESEARCH STUDY

Before taking part in this research, the researcher will talk to you, and will give you this consent form to read, as well as to clarify you where you might need clarity. You are cordially asked to sign the form after you have decided to take part in this project. I, ……………………………………. agree to participate in research titled "Evaluation of breeding practices and morphological characterization of donkeys in Blouberg Local Municipality, Limpopo province: Implication for the design of community-based breeding programme" in the Department of Agricultural Economics and Animal Production University of Limpopo.

Furthermore, I understand that:

1. My participation in this research is voluntary, and I will not gain monetary/ financial compensation for my participation. I may withdraw my participation in a case of discomfort, and my withdrawal will not affect my relationship with the researcher.

2. I have the right to not answer certain questions if I am uncomfortable. I understand that this participation is entirely voluntarily. I can withdraw my consent at any time with no penalty.
3. My response to the questions will be recorded at my permission. However, where I am not comfortable about recording my response, the researcher will have to write down my responses by him/herself.
4. Confidentiality and anonymity of records identifying you as a participant will be maintained by me and my learning institution, if necessary.
5. If you have any questions or concerns about participating in the interview or about contributing to this study, you may contact me or my supervisor on the numbers listed above.

____________________ ____________________ ______________

Participant Signature Date

(Full name)

I, Masixole Maswana, have clarified the scope of the research to the participant and explained his/ her rights concerning his/ her participation in the study. She/ He agrees to participate in this study.

Kind regards,

Researcher Signature Date
